# Social skills moderate pain sensitivity during acute psychosocial stress: an experimental study using the Trier Social Stress Test

**DOI:** 10.1186/s40101-026-00425-x

**Published:** 2026-02-27

**Authors:** Yoichi Tanaka, Nao Uchima, Wakana Ebisu, Yumiko Kawanishi, Riho Yamanaka, Ichika Wakatsuki, Kenta Hashimoto, Yuya Sakaguchi, Tsunehiro Otsuka, Ken Okutani, Daisuke Shimizu

**Affiliations:** Department of Occupational Therapy, School of Rehabilitation, Hyogo Medical University, 1-3-6 Minatojima, Chuo-Ku, Kobe, Hyogo 650-8530 Japan

**Keywords:** Social skills, Trait anxiety, Acute psychosocial stress, Pressure pain threshold, Trier Social Stress Test

## Abstract

**Background:**

This study examines whether acute psychosocial stress induced by the Trier Social Stress Test (TSST) alters pressure pain threshold (PPT) in healthy university students. Furthermore, we investigated how overall social skills and its subscales relate to stress-induced changes in PPT and whether trait anxiety moderates these relationships.

**Methods:**

A total of 34 healthy university students (14 males, 20 females; mean age = 21.2 ± 1.0 years) participated in the study. TSST was used to induce acute psychosocial stress. PPT, subjective stress, and autonomic activity (pulse wave amplitude and length) were assessed at three time points: pre, post, and 10-min recovery. Social skills were measured using the Adult Social Skills Scale, and trait anxiety was assessed using the STAI. Linear mixed-effects and regression models were applied to examine time effects and anxiety’s moderating role.

**Results:**

Subjective stress increased significantly immediately after TSST and returned to near baseline at the 10-min recovery. No significant changes were observed in pulse wave amplitude or length. A linear mixed-effects model revealed no significant main effect of time (*F*(2, 64) = 0.01, *p* = .99) or interaction between time and total social skills (*F*(2, 64) = 0.03, *p* = .97). A trend-level main effect of total social skills was observed (*F*(1, 31) = 3.28, *p* = .08), indicating that higher social skills were associated with greater PPT overall. Regression analyses of subscales revealed that only encoding skill exhibited a trend-level association with pre-to-post PPT change. A subsequent interaction model established a significant moderation by trait anxiety: the protective association of encoding skill with increased PPT was significant among low to average anxiety participants but not among high-anxiety participants.

**Conclusions:**

Individuals with higher social skills, particularly those with effective encoding abilities and lower trait anxiety, exhibited greater pain tolerance under stress, although acute social stress did not produce an overall change in PPT. These findings suggest that social communication competence may serve as a resilience factor in stress-related pain modulation, although elevated anxiety levels may attenuate such benefits.

## Introduction

Pain is a complex and subjective experience influenced not only by physiological mechanisms but also psychological and social contexts. Furthermore, individual differences in pain sensitivity have long been recognized and are thought to arise from interactions between biological, affective, and cognitive factors [1, 2]. Within this biopsychosocial framework, studies have repeatedly demonstrated that psychosocial variables such as anxiety, stress, and interpersonal relationships substantially modulate pain perception [3, 4]. Among psychosocial factors, social stress has recently gained attention as a potent modulator of pain. Social stress refers to psychological tension that arises from socially evaluative or interpersonal threat situations, such as public speaking, social exclusion, or criticism [5]. These experiences activate stress-responsive systems, including the hypothalamic–pituitary–adrenal (HPA) axis and sympathetic nervous system, thus leading to physiological and affective changes that can amplify pain [6, 7]. According to experimental studies, exposure to acute social stress, such as that during the Trier Social Stress Test (TSST), can alter pain thresholds and tolerance through neuroendocrine and emotional mechanisms [8]. Conversely, studies have confirmed that positive social interactions, such as empathy, belongingness, and perceived social support, buffer stress and reduce pain perception [9, 10]. Importantly, in these studies (refs. 8–10), the term pain refers specifically to experimentally induced physical pain, rather than to social or emotional pain. Against this background, it is important to distinguish social pain, which refers to distress arising from social exclusion or negative evaluation, from physical pain, which reflects nociceptive processing [11], a distinction that is clinically important because the present study targets modulation of physical pain under social-evaluative stress, rather than distress responses to social exclusion per se, thereby strengthening the rationale for using PPT as the primary outcome. Although conceptually distinct, social-evaluative stress has been suggested to influence physical pain processing via shared affective and central mechanisms, including threat appraisal and emotional regulation [12]. These processes are known to engage prefrontal–limbic circuits involved in top-down modulation of nociceptive input [13]. Accordingly, examining physical pain sensitivity under conditions of social stress provides a theoretically grounded framework for investigating how interpersonal traits, such as social skills, contribute to pain regulation beyond social distress per se. In the present study, physical pain sensitivity was assessed using the pressure pain threshold (PPT).

The capacity to establish and maintain such beneficial relationships is rooted in an individual’s social skills, which are defined as the ability to initiate, interpret, and express appropriate behaviors in social contexts [14]. Individuals with higher social skills tend to seek and receive more effective social support, regulate emotions more adaptively, and demonstrate better stress coping and well-being [15, 16]. Accordingly, those with stronger social skills may plausibly be more resilient to pain amplification during socially stressful situations.

However, the relationship between social skills and pain modulation may not be uniform across individuals. Moreover, research has identified trait anxiety, which is a stable tendency to perceive situations as threatening, as a key factor influencing both stress reactivity and pain regulation [4, 17]. High trait anxiety is associated with hyperactivation of the stress system and diminished endogenous pain inhibition, which suggests that anxiety could undermine the protective effects of social competence on pain modulation. Despite this theoretical relevance, the interaction between social skills and trait anxiety in predicting pain responses to social stress remains underexplored.

In the modern social environment, in which individuals are frequently exposed to evaluative and performance-related stressors, understanding how interpersonal abilities influence physical pain regulation has both theoretical and clinical importance. Building on these perspectives, the present study investigates the following: (1) whether acute social stress induced by the TSST alters physical pain sensitivity, as measured by the pressure pain threshold (PPT); (2) how the total and subscale dimensions of social skills are associated with stress-induced changes in PPT; and (3) whether trait anxiety moderates the relationship between social skills and pain modulation. Clarifying these relationships may provide novel insights into individual variability in pain reactivity and inform psychosocial approaches to stress-related pain management and potentially the prevention of stress-related pain exacerbation.

## Methods

### Ethics approval and consent to participate

The study employed a within-subject experimental design and was conducted in a quiet, temperature-controlled laboratory at Hyogo Medical University. The study protocol was approved by the Ethics Committee of Hyogo Medical University (approval number: 5034; 202508–010) and conducted in accordance with the Declaration of Helsinki. After providing a full explanation of the study procedures and objectives, we obtained written informed consent from all participants.

### Participants

Between July and September 2025, a total of 34 healthy university students (14 males and 20 females; mean age = 21.2 ± 1.0 years) participated in this study. Participants were recruited through on-campus advertisements and word-of-mouth at Hyogo Medical University (Nishinomiya, Japan). All participants were free of acute illness at the time of testing and were instructed to refrain from caffeine consumption and strenuous physical activity for 12 h prior to the experiment. The sample size was set a priori to approximately 30–40 participants, which is common among within-participant designs analyzed using linear mixed-effects models (LMMs). In repeated-measures settings, LMMs efficiently leverage multiple observations per participant, and a simulation-based power analysis is recommended to evaluate the detectable effects under realistic variance components. Following these methodological recommendations, our final sample of 34 participants, with three repeated measurements per session, was deemed adequate for the fixed-effect tests and interaction probing (simple slopes/Johnson–Neyman test).

### Procedure

Upon arrival, participants were seated in a quiet, temperature-controlled laboratory (approximately 25 °C). Participants first completed two self-administered questionnaires: Adult Social Skills Inventory (SSI) [18] and State-Trait Anxiety Inventory (STAI-Trait) [19]. Following a 10-min adaptation period, participants underwent the first assessment (pre, Measurement 1). Subjective stress, autonomic responses, and pain sensitivity were measured in the following fixed order:

1. Visual Analog Scale (VAS) for subjective stress intensity,
2. Pulse wave measurement (pulse wave amplitude and length),
3. PPT measured using a digital algometer.

Subsequently, the participants completed the TSST, consisting of two consecutive tasks:

- a 5-min speech task, in which participants delivered a self-introduction in front of two evaluators, and
- a 5-min arithmetic task, in which they continuously subtracted 13 from 2093 aloud.

Participants underwent the second assessment (post, Measurement 2) using the same VAS → pulse wave → PPT sequence immediately after the TSST. The third assessment (10-min recovery, Measurement 3) was conducted in the same order following a 10-min recovery period. The total duration of the experimental session was approximately 65 min for each participant.

#### Trier Social Stress Test (TSST)

The psychosocial stress induction followed the standardized TSST [20], which reliably elicits both subjective and physiological stress responses through social-evaluative and uncontrollable components [5, 6]. We used a modified individual version of the TSST. The participants were informed that they would perform two consecutive tasks in front of two evaluators. After a brief preparation period, they first delivered a 5-min self-introduction speech, which simulated a job interview situation. To enhance social-evaluative pressure, the evaluators maintained neutral facial expressions and refrained from providing any feedback throughout the task. Immediately afterward, participants performed a 5-min mental arithmetic task, during which they were instructed to continuously subtract 13 from 2093 aloud as quickly and accurately as possible. If an error occurred, they were instructed to restart from the initial number. Both tasks were conducted under observation by the same two evaluators seated at a desk, and participants were informed that their performance was being observed and appeared to be documented for evaluation by the evaluators. No audio or video recording was conducted, and no written records were retained. The entire stress procedure lasted approximately 10 min. Previous studies have validated this protocol as an effective laboratory method for inducing acute psychosocial stress and examining the associated changes in pain sensitivity and autonomic reactivity. Although this protocol differed slightly from the standard TSST in terms of evaluator number and experimental setting, it was designed to reliably induce subjective social-evaluative stress while maintaining feasibility in an individual laboratory context.

#### Psychological measures

##### Social Skills Self-Rating Scale for Adults

Social skills were assessed using the Social Skills Self-Rating Scale for Adults, which is a standardized instrument developed in Japan to evaluate interpersonal communication abilities in the Japanese cultural context [18]. This self-report questionnaire measures both the behavioral and expressive aspects of social interaction and consists of six subscales: start of relationships, decoding, claim/assertiveness, control of feelings, maintenance of relationships, and encoding. The scale is comprised of 35 items (including four reverse-scored items) rated on a 4-point Likert scale (1 = “strongly disagree” to 4 = “strongly agree”), with higher total scores reflecting greater social competence. Example items include: “You are friendly with anyone” (start of relationships); “You can read a person’s feelings from their facial expression” (decoding); “When your friend hurts your feelings, you clearly express how you feel” (claim/assertiveness); “Controlling your emotions is difficult” (control of feelings); “You behave appropriately depending on the situation” (maintenance of relationships); and “You openly express your feelings” (encoding). The scale has been widely used in Japanese psychological and clinical research and has demonstrated satisfactory internal consistency and construct validity [18].

##### State-Trait Anxiety Inventory (STAI-Trait)

Trait anxiety was assessed using the STAI [19] trait subscale, which measures the stable tendency to experience anxiety across situations. We used the Japanese validated version of the STAI. The STAI-Trait consists of 20 items rated on a 4-point Likert scale (1 = “almost never” to 4 = “almost always”), with higher scores indicating greater dispositional anxiety. With extensive application in both clinical and experimental research, the STAI demonstrates robust psychometric properties, including high internal consistency and test–retest reliability [21]. In our statistical analyses, the total STAI-Trait score was used as a continuous variable.

#### Subjective stress (VAS)

A 10-cm horizontal VAS, with anchors labeled “no stress” (0 cm) and “maximum stress imaginable” (10 cm) was used to evaluate subjective stress intensity. Participants were instructed to indicate their current stress level by marking a point along the aforementioned line in response to the question, “How much stress do you feel right now?” The distance from the left end of the scale (in centimeters) was recorded as the VAS score, with higher values indicating greater subjective stress intensity. The VAS is widely recognized as a sensitive and reliable method for assessing transient stress and emotional states in psychophysiological research. The primary advantage of the VAS lies in its simplicity and intuitive response format, thus allowing participants to provide rapid and direct self-assessments without verbal or cognitive burden [22, 23]. This makes the scale particularly suitable for repeated assessments during stress-inducing experimental paradigms, such as the TSST. In our study, VAS ratings were obtained at three time points: pre, post, and 10-min recovery.

#### Autonomic responses (pulse wave amplitude and length)

A photoplethysmographic finger volume pulse meter (BACS Detector, Japan) was used to assess autonomic responses. Participants were seated comfortably in a chair within a quiet laboratory maintained at approximately 25 °C. A probe was attached to the left index finger and pulse wave signals were recorded continuously for 60 s during each measurement phase. Participants were instructed to remain still and refrain from moving their hands during the recording. Then, two indices—pulse wave amplitude and length—were derived from the recorded waveform, which reflect peripheral vasomotor activity and the duration of one cardiac cycle, respectively. These photoplethysmographic indices have been widely used as non-invasive measures of autonomic nervous system activity and vascular tone in stress and psychophysiological research [24, 25].

#### Pressure pain threshold (PPT)

PPT was measured using a digital push–pull gauge (AIKOH Co., Japan). The participants were seated comfortably in a chair and pressure was applied perpendicularly to the nail of the right middle finger using a flat metal probe. The force was gradually increased at a constant rate until they verbally indicated the first sensation of pain. Participants were instructed to keep their hands still during the measurement to ensure stability. Each measurement was repeated three times at intervals of several seconds, and the mean value was used for analysis. The PPT assessment method has been widely used in experimental pain research and demonstrates good reliability for detecting changes in mechanical pain sensitivity [26, 27].

### Statistical analysis

R software (version 4.4.3; R Foundation for Statistical Computing, Vienna, Austria) and RStudio (version 2024.9.1) were used for all statistical analyses. Descriptive statistics were expressed as means ± standard deviations (SD). Statistical significance was set at *p* < 0.05 (two-tailed). First, to verify the effectiveness of stress induction by the TSST, subjective stress intensity (VAS) and autonomic indices (pulse wave amplitude and length) were analyzed using repeated-measures models, with time (pre, post, and 10-min recovery) as a within-subject factor. Next, an LMM was used to examine the effects of time, total social skills, and trait anxiety (STAI-Trait) on PPT. The model included time (three levels: pre, post, and 10-min recovery) as a within-subject fixed factor, *z*-standardized total social skill score, and *z*-standardized trait anxiety as fixed covariates, with a random intercept for each participant. To identify which components of social skills were most relevant to pain modulation, while controlling for trait anxiety, additional regression analyses were conducted for each of the six subscales: Start of relationships (SR), Control of feelings (CF), Encoding (EN), Decoding (DE), Maintenance of relationships (MR), and Claim/assertiveness (CL). Finally, a multiple regression model was fitted to examine the interaction between encoding skill and trait anxiety on changes in PPT (percentage change from pre to post). Simple slope analyses and the Johnson–Neyman technique were conducted to investigate significant interactions and identify regions of significance across the moderator range, respectively [28]. Following Nakagawa and Schielzeth, effect sizes for fixed effects in LMMs were calculated using the marginal and conditional *R*^2^ (variance explained by fixed factors or fixed and random factors, respectively) [29]. To examine potential multicollinearity, variance inflation factors (VIFs) were calculated for the regression models. All VIF values were low (range = 1.12–1.33), indicating that multicollinearity did not meaningfully affect the regression estimates. All plots were generated using the ggplot2 and interaction packages in R.

## Results

### Participants

A total of 34 healthy university students (14 males, 20 females; mean age = 21.2 ± 1.0 years) were included in the final analysis. For descriptive purposes, participants were divided into high and low social skills groups based on the median of the total social skill score (median = 97.5). No significant differences in age or sex distribution were observed between the groups (Table 1). The high-skill group exhibited significantly higher total and subscale scores on the SSI, particularly for SR, DE, MR, and CL (all *p* < 0.001). A trend toward higher EN scores was observed in the high-skill group (*p* = 0.07), whereas CF displayed no significant group differences (*p* = 0.82). Importantly, baseline PPT was significantly higher in the high-skill group (*p* < 0.05) than in the low-skill group, thereby suggesting greater pain tolerance before stress exposure. No significant differences were observed for trait anxiety (STAI-Trait) or baseline subjective stress intensity (VAS) (all *p* > 0.10). These findings confirm that, aside from the targeted social skill dimensions and PPT, the two groups were comparable in terms of demographic and psychological characteristics.

**Table 1:** Baseline characteristics of the participants (*n* = 34)

Variable	High skill (*n* = 17)	Low skill (*n* = 17)	*p* value
Gender (male/female)	9/8	8/9	.50
Age (years), mean (SD)	21.5 ± 0.6​	20.8 ± 1.2	.06
Outcome measures, mean (SD)			
Social skills total (SS)	104.6 ± 5.4​	84.8 ± 10.5	<.001
Start of relationships (SR)	24.0 ± 3.8	18.5 ± 4.1	<.001
Control of feelings (CF)	8.9 ± 1.6	8.7 ± 2.7	.82
Encoding (EN)	12.4 ± 2.3	11.7 ± 2.4	.07
Decoding (DE)	25.2 ± 2.9	19.6 ± 3.8	<.001
Maintenance of relationships (MR)	13.4 ± 1.1	11.4 ± 1.2	<.001
Claim (CL)	19.8 ± 2.9	15.2 ± 2.6	<.001
State–Trait Anxiety Inventory (STAI)–Trait subscale	45.1 ± 8.7	46.8 ± 8.7	.58
PPT (pre, kgf/cm^2^)	23.8 ± 9.2	17.5 ± 6.8	<.05
VAS (pre, 0–10)	1.56 ± 1.70	0.9 ± 1.1	.19

Values are mean ± SD unless otherwise indicated. *p*-values were obtained using independent t-tests (Welch’s correction when appropriate) or *χ*^2^ tests for categorical data. PPT (pre) represents PPT at the pre time point (prior to stress exposure)

Abbreviations: *SS *social skills, *SR* start of relationships, *CF* control of feelings, *EN* encoding, *DE *decoding, *MR* maintenance of relationships, *CL* claim, *STAI* State–Trait Anxiety Inventory (Trait subscale), *PPT* pressure pain threshold, *VAS* Visual Analog Scale

### Manipulation check

Subjective stress intensity (VAS) exhibited a significant main effect of time, *χ*^2^(2) = 28.7, *p* < 0.001. VAS scores markedly increased from pre (1.2 ± 1.4) to post (5.0 ± 2.4, *p* < 0.001) and decreased at 10-min recovery (1.7 ± 1.4, *p* < 0.001). No significant difference was observed between pre and 10-min recovery (*p* = 0.20). Thus, the TSST is confirmed to have successfully induced a transient increase in subjective stress that returned toward baseline within 10 min. By contrast, no significant time effects were observed for physiological indices. Pulse wave amplitude remained stable across pre, post, and 10-min recovery (*F*(2, 54) = 1.40, *p* = 0.255), and pulse wave length similarly exhibited no significant variation across time points (*F*(2, 54) = 0.78,* p* = 0.466). Thus, the TSST elicited clear subjective stress responses, whereas autonomic measures did not display corresponding changes.

### Main analysis: linear mixed-effects model

This study adopted an LMM to examine the effects of time, social skill, and trait anxiety on PPT. The model included time (pre, post, and 10-min recovery), *z*-standardized total social skill score, and trait anxiety (STAI) as fixed effects, with a random intercept for each participant. No significant main effect of time was observed (*F*(2, 64) = 0.006, *p* = 0.994) indicating that PPT did not significantly change across pre, post, and 10-min recovery. In addition, no significant interaction was observed between time and social skills (*F*(2, 64) = 0.032, *p* = 0.969). However, a trend-level main effect of total social skills was observed (*F*(1, 31) = 3.28, *p* = 0.08), which suggests that participants with higher social skills tended to exhibit higher PPT values overall, thus reflecting greater pain tolerance, irrespective of time. Trait anxiety (STAI) was not a significant predictor of PPT (*p* = 0.75). Collectively, these results indicate that while PPT did not fluctuate across the experimental session, individuals with higher social skills tended to maintain higher pain thresholds throughout the experimental period.

### Subscale analysis of social skills

To identify which components of social skills were most relevant to pain modulation, each of the six subscales (SR, CF, EN, DE, MR, and CL) was entered separately into regression analyses, controlling for trait anxiety (STAI-Trait). As summarized in Table 2, encoding skill exhibited a trend-level association with changes in PPT from pre to post (*β* = –5.87, *p* = 0.09), which suggests that participants with better encoding ability tended to exhibit a greater increase in pain tolerance following stress exposure. No other subscales were significantly associated with PPT change (all *p* > 0.25). According to these findings, the ability to express emotions and thoughts effectively in social interactions (encoding skill) may play a distinctive role in modulating pain sensitivity under psychosocial stress, whereas other interpersonal abilities such as decoding or assertiveness were not related to pain threshold changes.

**Table 2:** Regression coefficients for social skill subscales predicting percentage change in pressure pain threshold (pre to post)

Predictor (social skill subscale)	Estimate (β)	SE	*t* value	*p* value
Start of relationships (SR)	− 2.80	3.74	− 0.75	.46
Control of feelings (CF)	2.28	3.56	0.64	.53
Encoding (EN)	− 5.87	3.38	− 1.73	.09
Decoding (DE)	2.60	3.60	0.72	.48
Maintenance of relationships (MR)	3.49	3.49	1.00	.33
Claim (CL)	4.04	3.47	1.16	.25

Results are from separate linear regression models predicting the percentage change in pressure pain threshold (pre to post), with each subscale *z*-standardized and trait anxiety (STAI) included as a covariate. *n* = 34

### Interaction between encoding skill and trait anxiety

**Fig. 1:**
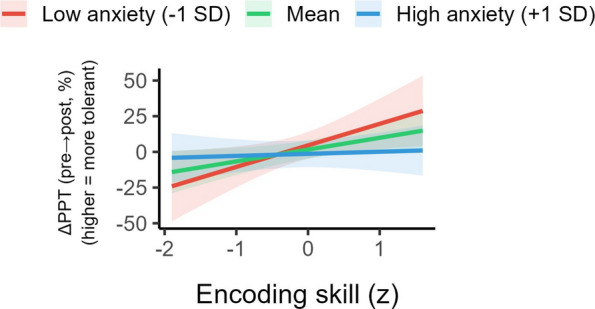
Interaction between encoding skill and trait anxiety on changes in pressure pain threshold (ΔPPT).. Lines represent model-predicted percentage changes in PPT from pre to post at low (− 1 SD; red), mean (green), and high (+ 1 SD; blue) levels of trait anxiety. Shaded areas indicate ± 1 SE of the predicted values. Greater ΔPPT reflects increased pain tolerance following stress

To further examine whether encoding skill interacts with trait anxiety in predicting pain modulation under stress, we conducted a multiple regression analysis using the *z*-standardized encoding score (SS_encode_z), trait anxiety (STAI_z), and their interaction term as predictors of the percentage change in PPT from pre to post. A significant interaction was observed between encoding skill and trait anxiety (*p* < 0.05; Fig. 1). Simple slope analysis revealed that encoding skill significantly predicted greater increases in PPT (i.e., reduced pain sensitivity) among participants with low-to-average anxiety (*p* = 0.03), but not among those with high anxiety (*p* = 0.74). The Johnson–Neyman analysis further established that this effect was significant when STAI_z values were between − 2.21 and 0.21, which suggests that the buffering effect of encoding skill on stress-induced pain sensitivity was limited to individuals with low or average levels of trait anxiety. According to our findings, the adaptive role of encoding skill in pain modulation depends on the individual’s anxiety level. Specifically, those who can effectively express emotions and maintain clear communication appear more resilient to pain amplification under psychosocial stress; however, this advantage diminishes as anxiety increases.

## Discussion

The present study investigated the effects of acute psychosocial stress on pain sensitivity, as well as how individual differences in social skills and trait anxiety contribute to pain modulation under stress. Contrary to our first hypothesis, despite a clear increase in subjective stress ratings, PPT did not significantly change following the TSST. However, a trend-level association was observed between total social skills and overall PPT, and encoding skill, which is a subcomponent reflecting expressive communication ability, was marginally associated with stress-related changes in PPT. Furthermore, an interaction between encoding skill and trait anxiety revealed that this protective effect was evident only among participants with low to moderate anxiety levels. Our findings highlight the nuanced roles of interpersonal and emotional factors in modulating pain under social stress.

### Effects of acute social stress on pain sensitivity

Previous studies have reported inconsistent effects of acute psychosocial stress on pain sensitivity, ranging from stress-induced analgesia to hyperalgesia, depending on the type and intensity of the stressor, as well as individual characteristics [30, 31]. In the present study, although subjective stress increased sharply immediately after the TSST, pressure pain threshold (PPT) remained stable across measurement points. One possible explanation is that the intensity of the social-evaluative stress induced by the modified TSST was insufficient to elicit detectable changes in mechanical pain sensitivity among healthy young adults. Although the TSST is well established as a reliable paradigm for inducing psychosocial stress, the present protocol differed from the original TSST protocol in several respects. Nevertheless, it retained the core components that induce social-evaluative stress (i.e., public speaking and mental arithmetic under evaluative observation), while the evaluative context was simplified. Specifically, the panel size was smaller (two evaluators rather than the three-person selection committee described in the original TSST protocol), and we did not implement the formal audio/video recording set-up and subsequent behavioral analysis described in the original TSST protocol. Despite these modifications, the evaluators maintained a neutral demeanor, and participants were led to believe that their performance was being evaluated, which is a key element for eliciting social-evaluative threat. Therefore, the present procedure is considered sufficient to induce acute psychosocial stress while maintaining feasibility in an individual laboratory setting. However, these procedural characteristics may have attenuated the level of uncontrollability and social-evaluative threat, which are considered critical components for robust activation of physiological stress responses. While the modified TSST was effective in eliciting a pronounced subjective stress response, it may not have been sufficient to strongly engage physiological stress pathways, such as hypothalamic–pituitary–adrenal (HPA) axis activation, that are more closely linked to stress-related changes in nociceptive processing. In addition, the absence of observable physiological changes may reflect limitations in the sensitivity of the autonomic indices used. Pulse wave amplitude and length provide indirect measures of peripheral vascular and autonomic activity, but such indices may be less sensitive to subtle or transient stress-related responses compared with other physiological markers, such as cortisol secretion or heart rate variability [20, 32]. Accordingly, the present findings do not preclude physiological stress activation per se, but rather suggest that such responses may not have been detectable using the selected indices under the current experimental conditions. It is also important to consider the characteristics of the present sample. With regard to trait anxiety, the mean STAI-Trait score (*M* = 45.9, SD = 8.6) was below levels typically considered indicative of elevated anxiety in clinical and applied research using the STAI [33]. In addition, the mean social skills score (*M* = 94.7, SD = 13.0) was comparable to values reported in the original scale development study of the Social Skills Self-Rating Scale for Adults conducted in a large sample of Japanese university students [18]. These findings indicate that the present participants did not represent an extreme or clinically vulnerable group in terms of anxiety or interpersonal competence, which may partly explain why acute social stress did not produce a robust change in PPT at the group level. Finally, previous research suggests that the effects of acute stress on pain are highly modulated by emotion regulation capacity, cognitive appraisal, and the availability of social coping resources [34, 35]. Thus, the absence of a group-level change in PPT in the present study may reflect compensatory psychological mechanisms that buffered stress-related pain amplification in this relatively healthy and resilient sample.

### Social skill and pain modulation

From a psychophysiological perspective, social skills may contribute to pain modulation by facilitating emotional and cognitive regulation processes involved in top-down control of nociceptive input. The trend-level main effect of total social skills suggests that individuals with higher interpersonal competence tended to exhibit higher pain thresholds across measurement points. This pattern may reflect a trait-like tendency in which socially skilled individuals are better able to maintain adaptive emotional states and limit negative affect that can amplify pain perception [36, 37]. Previous studies have reported associations between social skills and emotional clarity, cognitive reappraisal, and effective interpersonal coping [14, 38], which may contribute to a more stable psychological milieu. Accordingly, higher social skills may be associated with lower physiological arousal and reduced pain facilitation, particularly under relatively mild stress conditions. Among the six subscales, encoding skill—reflecting the ability to clearly and appropriately express one’s internal emotional states, intentions, and needs in social interactions—showed the strongest, albeit trend-level, association with stress-related changes in PPT. This finding may indicate that expressive communication is linked to more adaptive emotional regulation during social evaluation, potentially by facilitating emotional expression and self-regulatory processes. Individuals who can articulate their feelings more effectively may be less likely to internalize stress, which could attenuate the affective amplification of pain [39, 40]. Taken together, while these interpretations remain exploratory, the present findings suggest that social skills may be relevant to both trait-like differences in pain sensitivity and state-dependent variability in pain modulation under social stress.

### Moderating role of trait anxiety

The interaction between encoding skills and trait anxiety underscores the complexity of psychosocial influences on pain modulation. Although higher encoding skills predicted greater increases in PPT (reflecting reduced pain sensitivity) among participants with low to moderate anxiety, this was not the case among those with high anxiety. This suggests that high trait anxiety impairs the adaptive use of social and emotional competencies under stress. Individuals with elevated anxiety are more likely to interpret social evaluations as threatening, subsequently leading to hypervigilance, excessive self-monitoring, and difficulty in deploying regulatory strategies [17]. Consequently, anxiety may override the protective influence of social skills, resulting in diminished stress-buffering effects. This finding aligns with cognitive–affective models of pain, which posit that negative affect and heightened threat appraisal enhance pain perception via central sensitization and attentional bias [41, 42]. The results of our study extend this framework by demonstrating that the beneficial effects of interpersonal communication skills depend on the individual’s baseline anxiety level. From a clinical perspective, distinguishing physical pain from social pain is important when translating findings from social-evaluative stress paradigms to pain-related outcomes. Specifically, our results suggest that interpersonal communication skills may contribute to resilience in physical pain modulation under stress, which may have implications for psychosocial strategies aimed at mitigating stress-related pain vulnerability.

## Limitations and future directions

This study has several limitations. First, the sample consisted of healthy young adults, thereby limiting the generalizability to clinical or older populations. Second, only acute effects were examined; longitudinal or repeated-exposure designs may reveal cumulative or adaptive changes in pain regulation. Third, this study did not include physiological indices such as cortisol and heart rate variability, which could have provided mechanistic insights into stress–pain interactions. Accordingly, future research should incorporate multimodal stress measures and consider personality traits or coping styles as covariates.

## Conclusion

In summary, this study demonstrated that while acute social stress did not significantly alter mechanical pain threshold, individual differences in social skills, particularly encoding ability, played a meaningful role in stress-related pain modulation. Furthermore, this protective effect was evident only among individuals with lower anxiety, suggesting that effective emotional expression may have a buffering effect against pain amplification during social stress, whereas this advantage diminishes as anxiety increases. Finally, these findings highlight the need to consider both social and emotional traits in understanding stress-induced pain variability, and may inform psychosocial approaches to the management of stress-related pain and the prevention of stress-related pain exacerbation.

## Data Availability

The datasets generated and analyzed during the current study are available from the corresponding author on reasonable request.

## Abbreviations

CF: Control of feelings
CL: Claim/assertiveness
DE: Decoding
EN: Encoding
HPA: Hypothalamic–pituitary–adrenal
LMMs: Linear mixed-effects model
MR: Maintenance of relationships
PPT: Pressure pain threshold
SD: Standard deviations
SR: Start of relationships
SSI: Adult Social Skills Inventory
STAI-Trait: State-Trait Anxiety Inventory
TSST: Trier Social Stress Test
VAS: Visual Analog Scale
